# Using a Near-Infrared Spectrometer to Estimate the Age of *Anopheles* Mosquitoes Exposed to Pyrethroids

**DOI:** 10.1371/journal.pone.0090657

**Published:** 2014-03-04

**Authors:** Maggy T. Sikulu, Silas Majambere, Bakar O. Khatib, Abdullah S. Ali, Leon E. Hugo, Floyd E. Dowell

**Affiliations:** 1 Mosquito Control Laboratory, QIMR Berghofer Medical Research Institute, Brisbane, Queensland, Australia; 2 Environmental Futures Centre, Griffith University, Brisbane, Queensland, Australia; 3 Environmental Health and Ecological Sciences Thematic Group, Ifakara Health Institute, Ifakara, United Republic of Tanzania; 4 Vector Group, Liverpool School of Tropical Medicine, Liverpool, United Kingdom; 5 Zanzibar Malaria Control Programme, Ministry of Health and Social Welfare, Zanzibar, United Republic of Tanzania; 6 Engineering and Wind Erosion Research Unit, United States Department of Agriculture/Agricultural Research Services, Center for Grain and Animal Health Research, Manhattan, Kansas, United States of America; University of Queensland & CSIRO Ecosystem Sciences, Australia

## Abstract

We report on the accuracy of using near-infrared spectroscopy (NIRS) to predict the age of *Anopheles* mosquitoes reared from wild larvae and a mixed age-wild adult population collected from pit traps after exposure to pyrethroids. The mosquitoes reared from wild larvae were estimated as <7 or ≥7 d old with an overall accuracy of 79%. The age categories of *Anopheles* mosquitoes that were not exposed to the insecticide papers were predicted with 78% accuracy whereas the age categories of resistant, susceptible and mosquitoes exposed to control papers were predicted with 82%, 78% and 79% accuracy, respectively. The ages of 85% of the wild-collected mixed-age *Anopheles* were predicted by NIRS as ≤8 d for both susceptible and resistant groups. The age structure of wild-collected mosquitoes was not significantly different for the pyrethroid-susceptible and pyrethroid-resistant mosquitoes (P = 0.210). Based on these findings, NIRS chronological age estimation technique for *Anopheles* mosquitoes may be independent of insecticide exposure and the environmental conditions to which the mosquitoes are exposed.

## Introduction

Mosquito survival is critical to the transmission of the pathogens they carry. Malaria parasites take 9–14 d to mature inside the mosquito before they can be transmitted to a host. As such, only vectors that survive beyond the incubation period of the parasite become infectious [1]. Given that the main objective of existing vector control interventions such as the use of insecticide-treated nets and indoor residual spraying is to reduce mosquito lifespan to below the incubation period of the parasite [2], [3], the ability to determine the age of these vectors prior to and after implementation of a vector control program, is crucial to the assessment of the efficacy of these interventions.

The most common techniques used to estimate mosquito age structure are based on estimates of mosquito survival rates by their oviposition cycles. One of the key methods applied is the Detinova parity technique [4] which is a simple age grading technique that categorizes female mosquitoes into two age classes; nulliparous and parous. The second technique categorizes female mosquitoes according to the number of completed gonotrophic cycles [5]. However, the difficult and laborious dissections involved with this technique limit its use to a few experts working on only a small sample of a mosquito population. A relatively recent mosquito age grading methodology involves measuring changes in the abundance of cuticular hydrocarbons extracted from mosquito legs using mass spectrometry [6], [7]. This approach has been used to predict the ages of *Anopheles gambiae* into two age groups (≤2 d and >2 d) [8] and to predict the ages of *Anopheles farauti* into two age groups (<5 and ≥5 d) [9], but this technique is not cost-effective for large field trials, particularly in resource-limited areas. Another technique for analysing changes in the abundance of transcriptional profiles in the heads and thoraces of mosquitoes has been reported as the most accurate technique for predicting the age of *An. gambiae* [10]–[12]. However, earlier field-based experiments on uncaged *Aedes aegypti* using the transcriptional profiling age grading technique estimated the cost at US$7.50 per sample [13]. The cost and time involved could be major limitations to the applicability of this technique to processing large sample sizes required for large-scale field assessments.

Near-infrared spectroscopy (NIRS) is the most recent rapid and cost-effective age grading and species identification tool. It involves collecting spectrum absorbed from the heads and thoraces of mosquitoes and subsequent analysis of the spectrum for both age and species determination [14]. The technique has previously been used to simultaneously estimate ages and identify morphologically indistinguishable *An. gambiae* and *An. arabiensis* reared in the laboratory [14] and semi-field environment [15]. The method can also be applied to samples preserved in RNA*later* solution (Ambion, Austin, TX, USA) [16] and by other preservation techniques such as the use of desiccants, refrigeration, 70% ethanol and Carnoy's solution [17]. To date, NIRS has been used to predict mosquito age into two categories; <7 d old (an age group in which mosquitoes are too young to bear mature parasites) and ≥7 d old (an age group in which mosquitoes have survived long enough that they could be potentially infectious) with accuracies ranging from 78% to 90%. More recently, Ntamatungiro and colleagues indicated in their study that although the NIRS could not be relied upon for physiological age prediction of *An arabiensis*, its chronological age prediction ability is independent of the physiological status of the mosquitoes [18], meaning that NIRS can still be applied to predict the chronological age of a mosquito population with varying physiological age status. However, the performance of NIRS for age grading mosquitoes under a range of environmental exposures experienced by field mosquitoes has not been determined. In this present study, we examined the effect of insecticide exposure on the accuracy of NIRS mosquito age grading technique. The aims of this study were therefore 1) to evaluate the accuracy of the NIRS for predicting the age of *Anopheles* mosquitoes reared from wild larvae 2) to investigate whether exposure to insecticide affects the age prediction accuracy of *Anopheles* mosquitoes by NIRS and 3) to compare the age structure of pyrethroid-resistant and pyrethroid-susceptible adults collected from the field. The overall aim was to test whether NIRS can be applied as a technique for future evaluations of the efficacy of control interventions' in terms of removing potentially infectious or insecticide-resistant mosquitoes in the population.

## Materials and Methods

### Ethics statement

Ethics approval was obtained for routine blood feeding from the Zanzibar Malaria Control Programme (ZMCP) as part of the monitoring and evaluation program of malaria vectors. Ethics approvals were also obtained from the QIMR Berghofer Medical Research Institute (QIMR HREC980) and Griffith University (ENV/29/09/HREC). Written consent was obtained from all volunteers who were involved in blood feeding, and volunteers were given the right to refuse to participate or withdraw from the experiment at any time. Verbal consent was obtained from residents in Tumbe to dig pit traps around their homes for mosquito collection.

### Study site

This study was conducted at three ZMCP experimental sites, Tumbe, Chwale and Uwandani, on Pemba Island, Zanzibar, between October and November 2011. No specific permissions were required for these locations as they were within the ZMCP's approved research stations. This field study did not involve endangered or protected species. *Anopheles gambiae* s.l. (*Anopheles arabiensis, Anopheles merus and Anopheles gambiae* s.s.) are the main malaria vectors in these areas [19], [20].

### Larvae collection and rearing

Wild *Anopheles* spp. larvae and pupae were collected from sweet potato paddies, irrigation channels, grassy ditches and water logged areas of Tumbe, Chwale and Uwandani. The larvae and habitat water from the three sampling sites were pooled together in a plastic water trough measuring 35×15 cm in a room with naturally fluctuating humidity and ambient temperature. Additional food (Tetramin fish food flakes; Blacksburg, VA) was provided to larvae that were collected while still at the 1^st^ or 2^nd^ instar stages (10 g/1000 larvae). Pupae were placed in cages measuring 15×15×20 cm for a 24-hr emergence period, and thereafter, non-emerged pupae were removed and transferred to the next cage. Adult mosquitoes were fed on 10% glucose daily and on blood meals once every 7 d for 10 min on a human volunteer. The adults were provided with an oviposition site. Age samples required for NIRS calibration were collected at 1, 3, 5, 7, 9 and 14 d post emergence.

### Wild adult mosquitoes

All wild mosquitoes were collected in Tumbe from pit traps measuring 152×122×91 cm covered with coconut and banana leaves. Mosquitoes resting in pits were collected between 6 am and 9 am using an aspirator.

### Exposure of mosquito samples to insecticide

Wild mosquitoes were exposed to 0.05% lambda-cyhalothin as soon as they were collected from the field. A total of 335 wild mosquitoes were exposed [21]. Dead and surviving mosquitoes were preserved in RNA*later* for subsequent age grading using a near-infrared spectrometer [16].

### Spectra collection using the NIR spectrometer

All mosquitoes collected from Pemba were transferred to Ifakara Health Institute for NIRS analysis. Prior to scanning, residual RNA*later* was removed from the mosquito specimens using paper towels. At least 40 females at each age for both the lambda-cyhalothrin-exposed and unexposed laboratory cohorts, and 331 wild-collected mixed-age *Anopheles* mosquitoes were scanned using a LabSpec 5000 NIR spectrometer (ASD Inc, Boulder, CO), according to the procedure described by Mayagaya and colleagues [14]. A maximum of 20 mosquitoes were positioned ventral side up on a Spectralon plate. To collect the spectrum, the head and thorax of each mosquito was scanned one at a time under a 3-mm-bifurcated fiber-optic probe containing four collection fibers and 33 illumination fibers.

### Development of NIRS calibrations and data analysis

Calibrations were constructed that included mosquito age cohorts; that were not exposed to the assay at 1, 3, 5, 7, 9 and 14 d, mosquitoes that survived the 24-hr holding period after exposure to 0.05% lambda-cyhalothrin at 4, 8, and 13 d and mosquitoes that were exposed to control papers (oil-impregnated WHO assay papers) at 4, 8 and 13 d. Mosquitoes that died during the 24-hr holding period were excluded from the model. This calibration model was used to predict the age of the wild-caught mixed-age *Anopheles* mosquitoes and the mosquitoes that died after exposure to the insecticide. A total of eight factors developed from plots of prediction residual error sum of squares (PRESS) and regression coefficients were used in this model. The model included the portion of the NIRS spectrum from 500 nm–2350 nm. A Mann-Whitney U test was used to test whether the predictions were significantly different for resistant and susceptible wild-collected mosquitoes after exposure to 0.05% lambda-cyhalothrin. The ability of NIRS to differentiate three age groups for mosquitoes that survived or died after exposure to the assay was analysed using the Kruskal-Wallis test.

### Polymerase Chain Reaction to identify species

Using the alcohol precipitation method [22], wings or legs of adult mosquitoes were crushed in a test tube to extract DNA. Each of the sibling species of *An. gambiae* s.l. contains a unique sequence at the intergenic spacer region of ribosomal DNA that can be amplified for species identification [23]. A PCR master mix containing 1 U/µl Taq polymerase (New England Biolabs, Ipswich, MA,USA), 2 µl of 10 × KCL, 0.02 mM MgCL_2_, 2 mM dNTPs and 6.4 µl distilled water was added to the DNA extract; 10 pmole/µl of forward and reverse primers of the *An. gambiae* complex (*An. gambiae* s.s., *An. arabiensis*, *An. merus* and *Anopheles quadriannulatus* Theobald) and 10 pmole/µl of *An. funestus* specific primers were added to the PCR mix [23]. Samples of *An. gambiae* s.s. and *An. arabiensis* were used as positive controls in each batch of reactions, whereas distilled water was used as a negative control.

## Results

### Species identification

A total of 938 mosquitoes were analysed by PCR for species identification. The samples included both wild-caught and mosquitoes that were reared from wild collected larvae. After three attempts, the PCR assay successfully identified 39% (N = 365) of all the samples collected. Of the samples that were successfully amplified, 97% were *An. arabiensis* and the remainder were *An. merus*.

### NIRS age prediction of resistant and susceptible *Anopheles* spp reared from wild larvae

**Figure 1:**
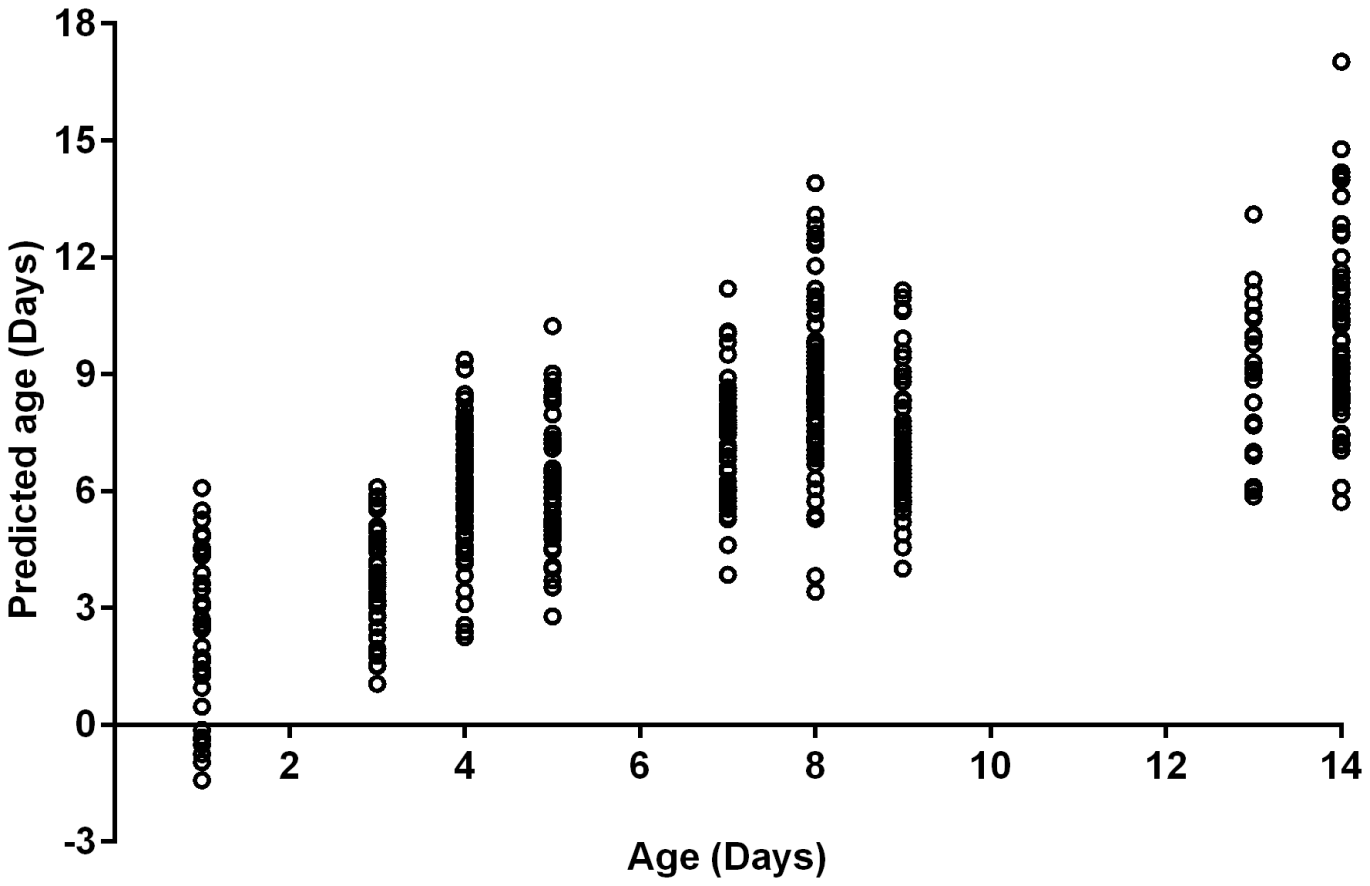
NIRS age prediction of individual female laboratory-reared *Anopheles* spp. The specimens include mosquitoes that were tested using the WHO resistance assay at 3, 7 and 12 d post emergence and sampled one day post pesticide exposure and unexposed mosquitoes sampled at 1, 3, 5, 7, 9 and 14 d post emergence.

The overall age estimation accuracy into <7 and ≥7 d age groups was 79% for all the mosquitoes, regardless of whether they had been exposed to the insecticide-treated papers. The age estimation accuracy for *Anopheles* mosquitoes that were not exposed to the insecticide-treated papers into <7 and ≥7 d age groups was 78%, whereas the age prediction accuracies into the two age groups for resistant and susceptible mosquitoes and mosquitoes used as controls were 82%, 78% and 79%, respectively (Table 1). The age prediction accuracy for mosquitoes that were 1, 3, 5, 7 and 9 d old varied between ±3 and ±5 d of the actual age, but the range increased to ±7 d for mosquitoes that were >9 d old (Table 2, Figure 1). The ages of mosquitoes at the extremes of the range tested (1–3 d old and 12–14 d old) were more accurately predicted into the two age categories (<7 or ≥7 d old).

**Table 1:** Percentage accuracy of the NIRS technique for predicting the age of mixed- *Anopheles* spp at each treatment level correctly as <7 or ≥7 d old.

Treatment group	%(N) correctly predicted as <7 d old	%(N) correctly predicted as ≥7 d old	Overall accuracy
Unexposed	86(122)	71(109)	78(231)
Resistant	76(51)	87(77)	82(128)
Susceptible	82(37)	75(56)	78(93)
Control	64(7)	84(31)	79(38)

**Table 2:** NIRS age prediction accuracy of mixed-*Anopheles* spp by age and treatment at different precision levels.

Actual age	Specimen condition	Range of prediction^b^	%(N) Predicted to within ±3 d of the actual age	%(N) Predicted correctly as <7 or ≥7 d
1	Unexposed	±4	90(37)	100(41)
3	Unexposed	±3	94(47)	100(50)
5	Unexposed	±5	94(48)	61(31)
7	Unexposed	±4	91(58)	64(41)
9	Unexposed	±5	79(37)	70(33)
14	Unexposed	±7	40(17)	100(45)
4	Resistant	±4	96(64)	76(51)
8	Resistant	±4	84(53)	86(54)
13	Resistant	±7	38(10)	82(22)
3.5^a^	Susceptible	±4	78(35)	82(37)
7.5^a^	Susceptible	±3	89(24)	56(15)
12.5^a^	Susceptible	±7	54(26)	88(43)
4	Control	±4	81.8(9)	64(7)
8	Control	±4	91(20)	74(17)
14	Control	±7	7(1)	88 (14)

Accuracy is shown for mosquitoes reared from wild larvae that were either untreated or treated with lambda-cyhalothrin. Mosquito categories that were treated include those that were resistant and those that were susceptible. Mosquitoes that were used as controls are also shown.

a Mosquitoes that died during the 24-hr holding period. Their actual ages are assumed to be 0.5 d older than the time of exposure. ^b^Range into which all mosquitoes in each age group were predicted.

**Figure 2:**
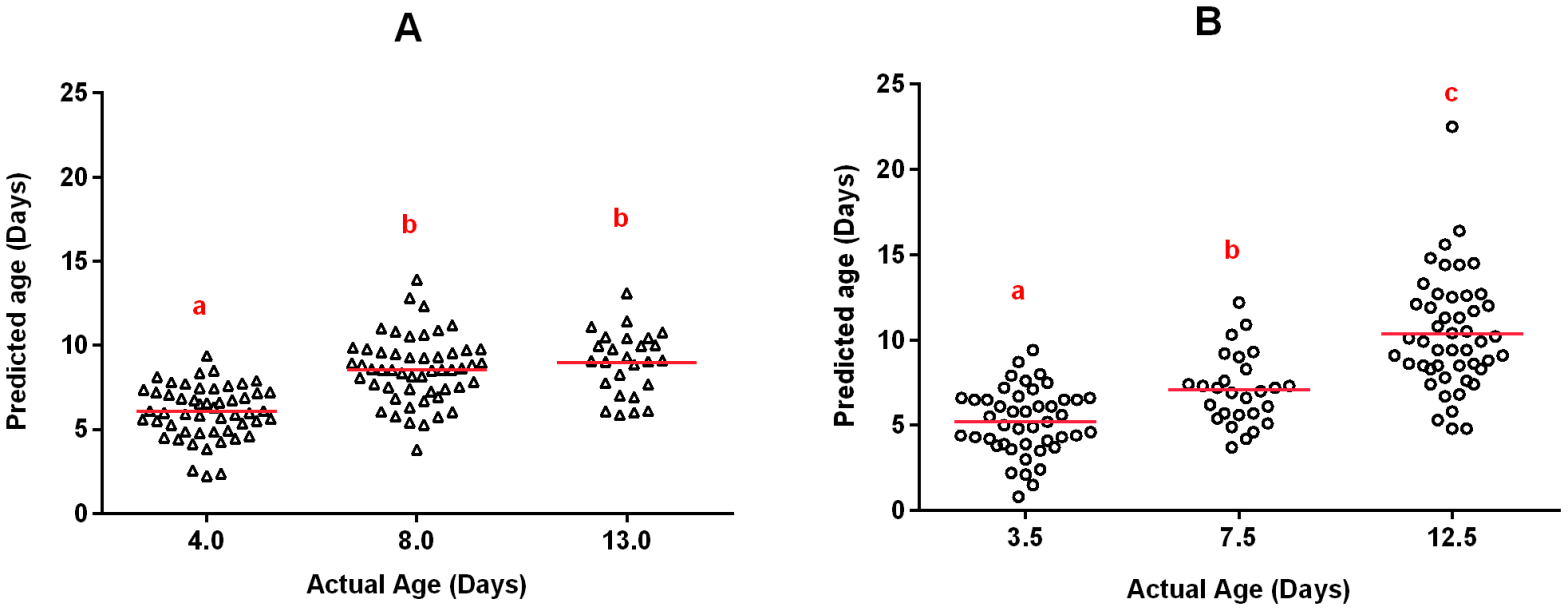
NIRS age predictions for A. resistant and B. susceptible laboratory reared *Anopheles* spp after 24-hr holding period post insecticide exposure. NIRS predictions that differ significantly between the different age groups at the 0.05 level are marked with a different letter. Mean age predictions are indicated by a red line.

The mean predicted ages (±SD) of the mosquitoes that survived the assay that were 4, 8 and 13 d old, were 6.0±1.5, 8.6±2.0 and 9.0±1.9 d, respectively, when predicted on a continuous scale (Figure 2A). The predicted values for the three age classes were significantly different from each other (Kruskal-Wallis; F = 71.27, d.f. = 2, P<0.001). Moreover, predictions for 4 d old mosquitoes were significantly different from predictions for both 8 d and 13 d old mosquitoes (P<0.05). However, no difference was observed between the age predictions for the 8 d old and 13 d old groups (P>0.05).

All laboratory-reared mosquitoes that died within the 24-hr holding period were assumed to be 0.5 d older than the time of insecticide exposure as the specific time of death was unknown. The mean age predictions (±SD) for 3.5, 7.5 and 12.5 d old groups on a continuous scale were 5.2±1.9, 7.1±2.1 and 10.4±3.3 d, respectively (Figure 2B), and these age predictions were significantly different from each other (F = 58.56, d.f. = 2, P<0.001).

### NIRS Age prediction of resistant and susceptible wild *Anopheles* spp

**Figure 3:**
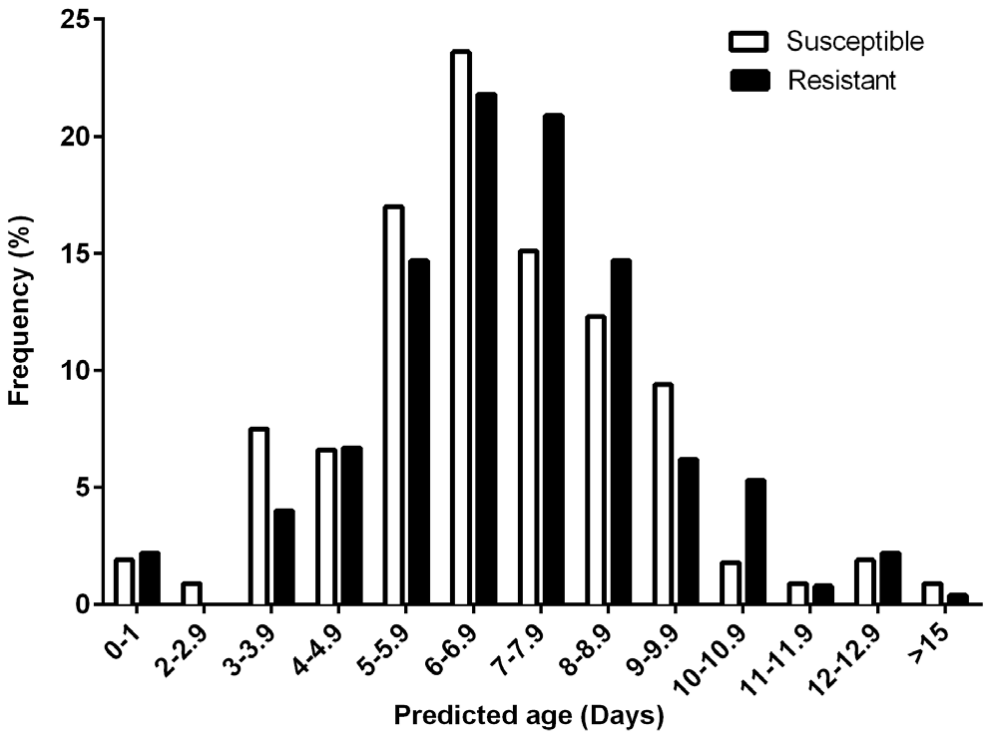
Frequency (%) age distribution of wild insecticide-resistant and susceptible mosquitoes, as predicted by NIRS, on a continuous age scale.

Wild mosquitoes were collected from pit traps over a period of 10 d and exposed to 0.05% lambda-cyhalothrin using the WHO resistance testing assay. No mortality were recorded in the control tubes therefore the test mortality was not adjusted. Of the total of 335 mosquitoes exposed to the assay, 69% survived the 24-hr holding period. NIRS was used to predict the age of 331 wild-collected mosquitoes. Of the wild-collected mosquitoes that survived the exposure to 0.05% lambda-cyhalothrin (N = 225), 72% were estimated to be between 5 and 8 d old, while only 3% of the mosquitoes that survived the treatment were estimated to be between the ages of 11 and 15 d old. The rest of the mosquitoes (25%) were estimated to be ≤4 d old. The age frequency of susceptible mosquitoes had a distribution similar to that of the resistant mosquitoes (Figure 3). Of the 106 mosquitoes that died after exposure to the assay, 65% were estimated to be between the ages of 5 and 8 d old, 13% were estimated to be ≥9 d old, and the rest were estimated to be ≤4 d old. In addition, 90% of all the wild mosquitoes that were found blood-fed during collection were estimated to be ≥5 d old. A comparisons of the estimated age frequencies of resistant and susceptible mosquitoes conducted using Mann-Whitney U test indicated that the age distributions of the two groups were not significantly different (P = 0.210).

## Discussion

In this investigation, the age prediction accuracy of a novel mosquito age grading technology, NIRS, was tested on *Anopheles* mosquitoes reared from wild larvae, mosquitoes exposed to pyrethroids and on wild collected adults to differentiate the age structures of pyrethroid-susceptible and pyrethroid-resistant mosquitoes. Using NIRS, the ages of adult *Anopheles* spp reared from wild larvae were predicted into <7 d or ≥7 d old categories with an overall accuracy of 79% by cross-validation and prediction analyses, regardless of whether the mosquitoes had been exposed to insecticide treated papers. However, neither species identity nor whether mosquitoes were exposed to the insecticide-treated papers or the control papers changed the prediction accuracy of NIRS. The NIRS age prediction accuracies (78–82%) obtained in this study for various treatments, are comparable to previously reported accuracies i.e., 80–83% accuracy for laboratory-reared fresh specimens [14], [16], 84% for laboratory-reared mosquitoes maintained in a semi-field environment [15] and 90% for RNA*later*-preserved laboratory-reared *An. gambiae* s.s and *An. arabiensis* mosquitoes [16], [17]. Recent reports indicate that the physiological age of *An. arabiensis* has no effect on the accuracy of chronological age prediction using NIRS [18]. The consistency of the accuracy of NIRS observed in several studies conducted using physiologically indistinct samples and samples maintained under various environmental conditions indicates its robustness as an age prediction tool.

The overall mortality of the mixed-age wild-caught *Anopheles* adults exposed to lambda-cyhalothrin was determined to be only 31%, confirming a high level of insecticide resistance in the population. Our results are consistent with those of a previous study conducted on Zanzibar Island where authors recorded an overall mortality of 32% for mixed-age wild DDT-resistant *Anopheles* mosquitoes [24]. It has been demonstrated in previous studies that insecticide resistance decreases with age in mosquitoes [24]–[27]. This difference presented a unique opportunity to test whether the NIRS age grading technology could detect differences in the age structures of resistant and susceptible mosquitoes collected as adults from a wild population. However, no difference was detected between the estimated age structures of resistant and susceptible wild-collected mosquitoes after exposure to lambda-cyhalothrin. Mosquitoes predicted to be ≤8 d old were the most frequently sampled for both the susceptible and the resistant groups. The wild adults were collected exactly three weeks after two weeks of heavy rainfall in this area. It is therefore possible that there was a large emergence of mosquitoes one week following the rainfall event, which might have created a pulse of mosquitoes that were ≤8 d old in the third week after the rainfall. Alternatively, the estimated age distribution could indicate a bias in pit trap collections towards females that are either nulliparous (2–5 d old) or those undergoing their first gonotrophic cycle (4-7 d old) as previously reported by Giles and his colleagues who compared the physiological and the chronological ages of *An. gambiae* and *An. funestus* in Tanzania [28]. The possibility that a majority of the samples collected from the wild belonged to ≤8 d age category is also reinforced by the 69% level of resistance observed. This level of resistance is more similar to the 44–72% resistance level observed for 3 to 5 d old *An. gambiae* s.l. [25], 84% resistance level observed for 4 to 6 d old unidentified *An. gambiae* s.l. population and 64% resistance level observed for *An. gambiae* s.s. [24] than to the lower resistance level observed for older insecticide resistant mosquitoes [24], [25]. Additionally, the close proximity of this particular adult sampling site to a large larval breeding site and residents' homes makes it a potential resting site for both newly emerged and blood-fed mosquitoes.

After several attempts to identify the anophelines by PCR using the method described by Scott and colleagues [23], we were unable to amplify rDNA fragments from the diagnostic Intergenic Spacer Region for a majority of the specimens collected. Based on the successful PCR reactions, *An. arabiensis* was the major species collected (97% from pit traps and 98% from mosquitoes reared from wild larvae) while *An. merus* was the only other species identified. Nonetheless, this study is consistent with recent findings from a study also conducted on Pemba Island in which collections consisted of 97% *An. arabiensis* and 3% *An. merus*. Furthermore, *An. gambiae* s.s. was absent from the specimens collected and 6% of their samples could not be amplified by PCR [20]. Failure to amplify ribosomal DNA from the majority of specimens collected on Pemba Island using a standard species identification test, could be due to technical errors occurring while performing PCR analysis or collection biases of pit trapping and our larval collections against *An. gambiae* s.l. sibling species that have been successfully identified previously. Alternatively, a tremendous success has been achieved in controlling *An. gambiae* s.s. following the introduction of LLNs on the island six years ago [29]. This study therefore provides grounds for more investigation regarding malaria species composition on the island.

Nonetheless, the success of NIRS to predict ages of mosquitoes in this sample suggest that species identity may not be important to successful age grading of *Anopheles* when using NIRS. Therefore, NIRS may be a broadly applicable technology suited to age grading *Anopheles* spp under a wide range of environmental conditions or those with prior exposure to insecticides. However, for the NIRS technology to be incorporated into routine monitoring and evaluation programs, a detailed study is recommended to validate its accuracy relative to traditional age grading techniques such as parity dissections or dissections conducted to determine the number of gonotrophic cycles a mosquito has undergone. Such study would require the development of a simplified field-based NIRS protocol for routine monitoring of national and international vector control programs.

On this occasion, NIRS was not attempted for the differentiation of sibling species collected as it has only previously been calibrated to differentiate *An. gambiae* from *An. arabiensis* where only these two sibling species co-exist [14], [15]. Historical reports indicate the presence of more than two sibling species of the *An. gambiae* s.l. in the current study area [19], [20], [24]. Future studies should therefore investigate the ability of NIRS to differentiate among three or more sibling species in an area where they occur sympatric with each other. Such studies would require development of new calibration models to incorporate the additional species.

In summary, these findings indicate that NIRS is a robust age prediction method for *Anopheles* mosquitoes. Future application of the technique as a monitoring and evaluation tool for malaria vector control interventions is envisaged.

## References

[1] Beier C (1998) Malaria parasite development in mosquitoes. Annu Rev Entomol 43: 519–543. 9444756 10.1146/annurev.ento.43.1.519

[2] Robert V, Carnevale P (1991) Influence of deltamethrin treatment of bed nets on malaria transmission in the Kou valley, Burkina Faso. Bull World Health Organ 69: 735–740. 1786622 PMC2393314

[3] Magesa SM, Wilkes TJ, Mnzava AEP, Njunwa KJ, Myamba J, et al (1991) Trial of pyrethroid impregnated bednets in an area of Tanzania holoendemic for malaria Part 2. Effects on the malaria vector population. Acta Trop 49: 97–108. 1680284 10.1016/0001-706x(91)90057-q

[4] Detinova T (1962) Age-grouping methods in Diptera of medical importance, with special reference to some vectors of malaria. Monogr Ser World Health Organ 47: 13–191. 13885800

[5] Polovodova VP (1949) The determination of the physiological age of female *Anopheles* by number of gonotrophic cycles completed. Med Parazitol Parazitar Bolezni 18: 352–355.

[6] Desena ML, Edman JD, Clark JM, Symington SB, Scott TW (1999) *Aedes aegypti* (Diptera: Culicidae) age determination by cuticular hydrocarbon analysis of female legs. J Med Entomol 36: 824–830. 10593086 10.1093/jmedent/36.6.824

[7] Desena ML, Clark JM, Edman JD, Symington SB, Scott TW, et al (1999) Potential for aging female *Aedes aegypti* (Diptera: Culicidae) by gas chromatographic analysis of cuticular hydrocarbons, including a field evaluation. J Med Entomol 36: 811–823. 10593085 10.1093/jmedent/36.6.811

[8] Caputo B, Dani R, Horne L, Petrarca V, Turillazzi S, et al (2005) Identification and composition of cuticular hydrocarbons of the major Afrotropical malaria vector *Anopheles gambiae* s.s. (Diptera: Culicidae): analysis of sexual dimorphism and age-related changes. J Mass Spectrom 40: 1595–1604. 16320293 10.1002/jms.961

[9] Hugo LE, Kay BH, Eaglesham GK, Holling N, Ryan PA (2006) Investigation of cuticular hydrocarbons for determining the age and survivorship of Australian mosquitoes. Am J Trop Med Hyg 74: 462–474. 16525108

[10] Cook PE, Sinkins SP (2010) Transcriptional profiling of *Anopheles gambiae* mosquitoes for adult age estimation. Insect Mol Biol doi: 10.1111/j.1365-2583.2010.01034.x. 10.1111/j.1365-2583.2010.01034.x PMC2998705 20695922

[11] Wang M-H, Marinotti O, James AA, Walker E, Githure J, et al (2010) Genome-wide patterns of gene expression during aging in the African malaria vector *Anopheles gambiae* . PLoS ONE 5: e13359. 20967211 10.1371/journal.pone.0013359 PMC2954169

[12] Wang M-H, Marinotti O, Zhong D, James AA, Walker E, et al (2013) Gene expression-based biomarkers for *Anopheles gambiae* age grading. PLoS ONE 8: e69439. 23936017 10.1371/journal.pone.0069439 PMC3720620

[13] Hugo E, Peter E, Petrina H, Luke P, Brian H, et al (2010) Field validation of a transcriptional assay for the prediction of age of uncaged *Aedes aegypti* mosquitoes in northern Australia. PLoS Negl Trop Dis 4: e608. 20186322 10.1371/journal.pntd.0000608 PMC2826399

[14] Mayagaya VS, Michel K, Benedict MQ, Killeen GF, Wirtz RA, et al (2009) Non-destructive determination of age and species of *Anopheles gambiae* s.l. Using near-infrared spectroscopy. Am J Trop Med Hyg 81: 622–630. 19815877 10.4269/ajtmh.2009.09-0192

[15] Sikulu M, Killeen G, Hugo L, Ryan P, Dowell K, et al (2010) Near-infrared spectroscopy as a complementary age grading and species identification tool for African malaria vectors. Parasit Vectors 3: 49. 20525305 10.1186/1756-3305-3-49 PMC2902455

[16] Sikulu M, Dowell K, Hugo L, Wirtz R, Michel K, et al (2011) Evaluating RNAlater as a preservative for using near-infrared spectroscopy to predict *Anopheles gambiae* age and species. Malar J 10: 186. 21740582 10.1186/1475-2875-10-186 PMC3157445

[17] Dowell FE, Noutcha AEM, Michel K (2011) The effect of preservation methods on predicting mosquito age by near Infrared spectroscopy. Am J Trop Med Hyg 85: 1093–1096. 22144450 10.4269/ajtmh.2011.11-0438 PMC3225158

[18] Ntamatungiro A, Mayagaya V, Rieben S, Moore S, Dowell F, et al (2013) The influence of physiological status on age prediction of *Anopheles arabiensis* using near infra-red spectroscopy. Parasit Vectors 6: 298. 24499515 10.1186/1756-3305-6-298 PMC3852719

[19] Mnzava AE, Kilama WL (1986) Observations on the distribution of the *Anopheles gambiae* complex in Tanzania. Acta Trop 43: 277–282. 2877554

[20] Haji K, Khatib B, Smith S, Ali A, Devine G, et al (2013) Challenges for malaria elimination in Zanzibar: pyrethroid resistance in malaria vectors and poor performance of long-lasting insecticide nets. Parasit Vectors 6: 82. 23537463 10.1186/1756-3305-6-82 PMC3639098

[21] WHO (1998) Techniques to detect Insecticide resistance mechanisms (field and laboratory manual).Geneva. Bull World Health Organ WHO/CDS/CPC/MAL/986.

[22] Collins FH, Mendez MA, Rasmussen MO, Mehaffey PC, Besansky NJ, et al (1987) A ribosomal RNA gene probe differentiates member species of the *Anopheles gambiae* complex. Am J Trop Med Hyg 37: 37–41. 2886070 10.4269/ajtmh.1987.37.37

[23] Scott J, Brogdon W, Collins F (1993) Identification of single specimens of the *Anopheles gambiae* complex by the polymerase chain reaction. Am J Trop Med Hyg 49: 520–529. 8214283 10.4269/ajtmh.1993.49.520

[24] Lines JD, Nassor NS (1991) DDT resistance in *Anopheles gambiae* declines with mosquito age. Med Vet Entomol 5: 261–265. 1768918 10.1111/j.1365-2915.1991.tb00550.x

[25] Jones C, Sanou A, Guelbeogo W, Sagnon NF, Johnson P, et al (2012) Aging partially restores the efficacy of malaria vector control in insecticide-resistant populations of *Anopheles gambiae* s.l. from Burkina Faso. Malar J 11: 24. 22269002 10.1186/1475-2875-11-24 PMC3312828

[26] Rajatileka S, Burhani J, Ranson H (2011) Mosquito age and susceptibility to insecticides. T Roy Soc Trop Med 105: 247–253. 10.1016/j.trstmh.2011.01.009 21353689

[27] Glunt KD, Thomas MB, Read AF (2011) The effects of age, exposure history and malaria infection on the susceptibility of *Anopheles* mosquitoes to low concentrations of pyrethroid. PLoS ONE 6: e24968. 21966392 10.1371/journal.pone.0024968 PMC3178580

[28] Gillies MT, Wilkes TJ (1965) A study of the age composition of *Anopheles gambiae* Giles and *A. funestus* Giles in north-eastern Tanzania. Bull Entomol Res 56: 237–262. 5854754 10.1017/s0007485300056339

[29] Bhattarai A, Ali AS, Kachur SP, Mårtensson A, Abbas AK, et al (2007) Impact of Artemisinin-based combination therapy and insecticide-treated nets on malaria burden in Zanzibar. PLoS Med 4: e309. 17988171 10.1371/journal.pmed.0040309 PMC2062481

